# Cognitive flexibility in autism: Evidence from young autistic children

**DOI:** 10.1002/aur.2828

**Published:** 2022-10-04

**Authors:** Maria Andreou, Kostas Konstantopoulos, Eleni Peristeri

**Affiliations:** ^1^ Department of Speech and Language Therapy University of Peloponnese Kalamata Greece; ^2^ School of English Aristotle University of Thessaloniki Thessaloniki Greece

**Keywords:** autism, children's color trails test, cognitive flexibility, fluency

## Abstract

We examined the cognitive flexibility performance of young autistic children and a group of neurotypical peers. Thirty‐six autistic children (72–83 months) and 200 age‐matched typically‐developing children were assessed on the Children's Color Trails Test (CCTT), a semantic and a phonemic verbal fluency task. The results showed that the autistic children performed worse than their neurotypical peers in the switching component of the CCTT. In the fluency tests, the autistic group generated overall fewer word items than their neurotypical peers, however, their poorer performance was driven by specific linguistic stimuli in the fluency tasks. The findings suggest that cognitive flexibility for the autistic children was affected in the nonverbal CCTT only, while poor performance in semantic and phonemic fluency seemed to be inherent to the language properties of the verbal fluency tasks.

## Lay Summary

The current study aims to determine whether the Children's Color Trails Test (CCTT) and verbal (semantic and phonemic) fluency tasks are appropriate to assess cognitive flexibility in young autistic children. The overall findings showed poor performance for the autistic children in the CCTT. On the other hand, verbal fluency performance seemed to be driven by language features specific to the semantic and the phonemic fluency tasks rather than by difficulties in cognitive flexibility.

## INTRODUCTION

Cognitive flexibility is a common aspect of executive functions and refers to the ability to adapt cognitive behavior in response to changing concepts, tasks or types of information (Ionescu, 2012). Although cognitive flexibility is an “umbrella” term that incorporates a set of executive function skills, shifting has been treated as being synonymous with this cognitive skill, since both involve the ability to switch flexibly between mental states and take multiple simultaneous perspectives in response to the changing context (Vandierendonck et al., 2010). Decades of research have provided compelling evidence that autism is characterized by difficulties in cognitive flexibility (Fujino et al., 2019; Peristeri et al., 2020, 2021), but the nature and magnitude of these difficulties is unclear, due to assessments that may not accurately represent the potential of young autistic children (Kapp et al., 2013), as well as the varied ability levels of participants, complexity of tasks, and stimulus domains used (Landry & Al‐Taie, 2016; Memari et al., 2013). For instance, studies that have used performance‐based tasks (e.g., Wisconsin Card Sorting Task) to assess cognitive flexibility were more discordant about the presence of difficulties in autism than studies that used parental questionnaire‐based assessments (e.g., Behavioral Rating Inventory of Executive Function; see Landry & Al‐Taie, 2016 for a meta‐analysis). Difficulties in cognitive flexibility in autism have been mainly evident in attention‐shifting (Eigsti et al., 2008; Memari et al., 2013; Mostert‐Kerckhoffs et al., 2015), updating (Peristeri et al., 2020) and verbal dual‐task interference paradigms (Peristeri et al., 2021), and they have been linked to autistic children's increased susceptibility to perseverative thought and behavior. Difficulties in cognitive flexibility have also been key to explaining autistic school‐aged children's rigidity in judgment and decision making despite subsequent correction or cues denoting changing task conditions (Bos et al., 2019; D'Cruz et al., 2013); autistic individuals tend to perseverate on narrow topics and struggle with transitions or seeing novel relationships.

Most of the studies that have explored cognitive flexibility in autism conclude findings mainly based on comparisons of school‐aged autistic children against their typically‐developing (TD) peers, while studies that focus on the early expression of difficulties in flexibility in autism remain scarce. For instance, Kimhi et al. (2014) found that autistic preschool children exhibit poorer planning ability compared to a group of chronological‐ and mental age‐matched TD children. Furthermore, McEvoy et al. (1993) administered a battery of executive function tasks to preschool‐age autistic children, children with developmental delay, and TD children. According to the results, the autistic children were less able to flexibly change sets on a spatial reversal task, and exhibited more perseverative errors than the rest of the groups. Interestingly, Yerys et al. (2007) examined preschoolers with autism on flexibility measures and yielded no significant differences between the autistic group and peers with TD (see Friedman & Sterling, 2019 for a review in executive functions in autistic children).

The Children's Color Trails Test (CCTT) and verbal fluency, that is, the ability to retrieve members belonging to a specific category within a limited period of time, are two common neuropsychological tests used to assess cognitive flexibility in both clinical and typical school‐aged child populations (e.g., Koren et al., 2005; Leung & Zakzanis, 2014; Pastor‐Cerezuela et al., 2016; Stad et al., 2019; Zhao et al., 2013). While both tasks have been developed as reliable measures of cognitive flexibility competence in primary school‐aged (and older) children, the concurrent and predictive validity of these assessment measures at younger ages in autism has not been thoroughly examined. This information is needed to inform interpretation of assessment results in young autistic children with implications for improving study design as well as assessment and diagnostic practices for children under 7 years. In this paper, we sought to contribute to the knowledge regarding difficulties in cognitive flexibility in young autistic children by reporting findings from the CCTT, as well as from semantic and phonemic fluency tasks.

The CCTT has been employed as a representative measure of cognitive flexibility competence as it taps into prototypically flexible behaviors, such as the ability to shift between different tasks as a response to changing task demands (Cragg & Chevalier, 2012; Garcia‐Garcia et al., 2010). More specifically, in the CCTT individuals are first required to respond in a certain way with a set of stimuli by following one rule (i.e., connecting numbers in an ascending order; CCTT‐1), and are subsequently asked to follow another rule (i.e., connecting an alternating sequence of numbers and colors; CCTT‐2). These task‐switching requirements mirror the core features of cognitive flexibility as the individual needs to reconfigure mental sets to rapidly change from one task to another, which is cognitively more demanding as compared to the non‐shifting task condition. Besides cognitive flexibility, the CCTT also serves as a behavioral index of visual scanning and attention, since the individual needs to scan the visual display that contains distinct stimuli (i.e., numbers, different colors) to continuously identify the relevant ones, as well as psychomotor speed since numbers and colors in the switching version of the task need to be integrated rapidly (Blanco‐Gómez et al., 2015; Goldschmidt et al., 2019; Konstantopoulos et al., 2015).

A key merit of the CCTT is its simple instructions and time efficiency of administration, since its takes between 5 and 7 min in total (along with the instructions and the practice trials) to be completed. Also, the CCTT offers an advantage over other cognitive flexibility tests, due to its minimal cultural bias and language ability requirements (neither advanced vocabulary nor complex syntax in the instructions) so as not to burden language comprehension (Mok et al., 2008). Importantly, verbal ability requirements have been often found to impede the behavioral characterization of executive function abilities in young autistic children, while it is possible that response time variability in language‐mediated executive function tasks essentially measure verbal abilities rather than the children's switching competence itself (Akbar et al., 2013; Kleinhans et al., 2005; Russell et al., 1999). Despite these merits, the documentation of autistic children's performance in the CCTT has been scarce so far. To the best of our knowledge, previous research obtained CCTT performance data from a wide age range of autistic individuals, which might have masked the early expression of difficulties in cognitive flexibility in autistic children. Specifically, Han et al. (2011) found that autistic children with low IQ (<80) needed significantly more time to complete the CCTT relative to age‐matched autistic children with normal (>80) IQ scores, while in a follow‐up study the same authors found no switching cost difference between 8 and 17 year‐old autistic individuals and their neurotypical peers in completion time or accuracy (Han & Chan, 2017).This may be explained by the wide age range or/and the cognitive functioning profiles of the participants which have been scantly described in Han and Chan's (2017) follow‐up study.

Verbal fluency has been typically measured with two tasks, namely, semantic and phonemic fluency, which reflect the integrity of the semantic system (i.e., meaning‐based mental storage & organization of language), and the phonological lexicon (i.e. sound‐based mental storage & organization of language). Tests of semantic and phonemic fluency have been extensively used to measure important cognitive and language functions in children and adults, such as cognitive flexibility, monitoring and strategic search in the semantic system or/and phonological lexicon, respectively (Cretenet & Dru, 2009; Hurks et al., 2010; Koren et al., 2005). Traditional measures of verbal fluency tasks are ‘clustering’, that is, number of items in each cluster within phonemic or semantic subcategories, and ‘switching’, that is, number of switches between clusters of semantically or phonologically related items. Clustering is usually thought to result from the strategic search and automatic activation of words through the phonological lexicon and the semantic system of the individual. A cluster has been defined as two or more words sharing the same semantic subcategory (e.g., ‘cat’ and ‘dog’ belong to the pet animal cluster) or beginning with the same first two letters (e.g., ‘fan’ and ‘fat’) for the semantic and the phonemic fluency test, respectively. The switching component of fluency tasks, on the other hand, has been claimed to be a demanding cognitive process, since the participant needs to flexibly switch between two semantically or phonologically approximate subcategories (e.g., pets and farm animals in semantic fluency tasks) to facilitate lexical retrieval (Pastor‐Cerezuela et al., 2016; Unsworth et al., 2011). Number of switches equals the number of transitions between clusters. For example, the sequence ‘cat, dog; leopard, elephant; donkey, hen, pig’ in a semantic fluency test involves two switches—before leopard and after elephant. The enhanced cognitive flexibility demands of verbal fluency tests have been confirmed by many studies that have shown significant correlations between the total number of words generated in the fluency tasks, and tasks that assess cognitive mechanisms claimed to underpin the cognitive flexibility construct, such as inhibition (Fisk & Sharp, 2004; Henry et al., 2015), working memory (Azuma, 2004; Daneman, 1991), and updating (Aita et al., 2019; Shao et al., 2014).

Relatively few studies have assessed cognitive flexibility skills through verbal fluency tasks in autistic children. Begeer et al. (2014) have examined the cognitive abilities involved in the processes of clustering and switching during a verbal fluency task in autistic children and adolescents, and their neurotypical peers. The results demonstrated difficulties in the ability of switching in the autistic group, nevertheless the autistic participants tended to generate bigger clusters of lexical items than their neurotypical peers. Furthermore, Pastor‐Cerezuela et al.'s (2016) semantic fluency study with 5–8 year old children with and without autism revealed that the autistic children scored significantly lower in both the clustering and switching conditions as compared to the TD group, but better in clustering than in the switching component, which suggests cognitive flexibility difficulties. Chronological age in Pastor‐Cerezuela et al.'s (2016) study accounted for the variability in the autistic children's performance in the semantic fluency task, however, language proficiency was also found to play a significant role in the children's fluency performance.

In the current study, we sought to contribute to the knowledge regarding cognitive flexibility development in autism by investigating young autistic children's performance in the CCTT, as well as in semantic and phonemic fluency tasks in comparison to TD peers. If any of the tasks proves to be sensitive to cognitive flexibility difficulties in autism at an early age, it would enable the use of the same task paradigm(s) across wider age ranges, which would in turn help reveal a nuanced developmental trajectory in cognitive flexibility development in autism. Regarding the study's hypotheses, in line with previous studies using non‐verbal shifting tasks (Landry & Al‐Taie, 2016; Peristeri et al., 2020), we predicted that the young autistic children would exhibit greater switching costs than their TD peers in the component of the CCTT that required from children to join an alternating sequence of numbers and colors (i.e. the CCTT‐2). Regarding the verbal fluency tests, we predicted that the young autistic children would perform poorer than their TD peers, however, we hypothesized that this effect would be mediated by features specific to the language (semantic and phonemic) categories involved in the verbal fluency tests. As of yet, no study has incorporated the distinct features of semantic and phonemic categories of fluency tasks into our understanding of cognitive flexibility in autism, despite what is known about the role of semantic and phonemic information in strategic search of the lexicon (Cretenet & Dru, 2009; Hurks et al., 2010; Koren et al., 2005).

## METHODOLOGY

### Participants

The study included 236 children in total; 36 Greek‐speaking autistic (28 males) and 200 age‐, V(erbal) IQ‐, P(erformance) IQ‐, and socioeconomic status (SES)‐matched TD age‐matched children (104 males) ranging in age from 72 to 83 months. The autistic sample in the current study was characterized by a male preponderance with four to five times more males diagnosed than females. The autistic children were recruited from three public schools and two public diagnostic centers in Thessaly, central Greece. They had received a diagnosis of autism from a licensed child psychiatrist or developmental pediatrician according to the standard diagnostic criteria (DSM‐5; American Psychiatric Association, 2013). We confirmed the diagnosis of the autistic children using the Autism Diagnostic Interview‐Revised (ADI‐R; Rutter et al., 2003). At the group level, the autistic (and TD) children had a mean VIQ and PIQ score within the normal range (i.e., VIQ‐PIQ > 80, e.g., Bal et al., 2021; Botting & Conti‐Ramsden, 2003; Koyama et al., 2007; Siegel et al., 1996) as measured through the Greek version of the Wechsler Preschool and Primary Scale of Intelligence‐Third Edition (WPPSI‐III; standardized in Greek by Sideridis & Antoniou, 2015); independent‐samples t‐tests showed that there was no significant group difference in either PIQ (*p* = 0.549) or VIQ (*p* = 0.202). The VIQ and PIQ scores of the autistic children in the current study were within the normal intelligence range (≥80). Since the children did not face any cognitive developmental delay, they all attended the mainstream general kindergarten classroom rather than special classes with one‐to‐one educational support.

According to the parental reports, none of the autistic children that participated in the study had received speech and language therapy in the past. Data collection for the current study took place shortly after the children received their first diagnosis of autism. As the centers in the specific geographical region, that is, Thessaly, serve a diverse and economically disadvantaged population, children tend to receive a diagnosis rather late with more than half of the cases of autism (57%) being diagnosed in the 8–10 age group (Thomaidis et al., 2020). Typically developing children were recruited from kindergarten schools in Greece. The parents who provided written consent for the children's participation in the study completed a questionnaire with demographic information, where they reported no family history of learning disabilities or/and language/cognitive deficits for their child. Absence of language and cognitive difficulties was also confirmed by the kindergarten teachers' written reports on the children's academic performance and social functioning (see Konstantopoulos et al., 2015 for more details). We should note that kindergarten education in Greece obligatorily lasts 2 years (4 year olds through age six), and that all the children that have participated in the current study attended their second year. Preschool education in Greece insists on the importance of phonics instruction and grapho‐phonemic correspondences, since direct instruction on letter knowledge and phonological awareness is mandated by the national kindergarten curricula (Manolitsis et al., 2011; Stellakis, 2012). By age six, children in Greece are expected to have acquired all grapheme‐phoneme correspondences and other letter combination patterns, such as digraphs.

Details of participants' demographic characteristics, including chronological age, sex, VIQ and PIQ are presented in Table 1. We used independent‐samples *t*‐tests for numerical variables, and chi‐square tests (Yates correction) for gender.

**TABLE 1:** Demographic characteristics of TD children and autistic children

	Group means ranges		*Chi‐square*/*t* value	*p* value
	TD (*n* = 200)	Autism (*n* = 35)		
Sex (males/females)	104/96	28/8	8.225	0.003
Age (months; mean [SD])	78.7 (*2.9*) 72–83	80.4 (*4.5*) 72–87	0.890	0.104
Verbal IQ (mean [SD])	89.3 (*6.0*) 80–121	91.2 (*8.7*) 82–103	1.279	0.202
Performance IQ (mean [*SD*])	91.5 (*8.4*) 81–121	92.6 (*10.3*) 80–113	0.599	0.549
Socioeconomic status (maternal education in yeas)	10.2 (*3.3*) 5–18	9.7 (*3.6*) 6–18	0.679	0.498

### Materials

#### Children's color trails test

##### Stimuli and procedure

The CCTT test was administered to the participants according to the test guidelines (Llorente et al., 2003). The CCTT‐1 administration involved the connection of circled numbers (1–15) with a pencil on an A4 page (21 cm × 28 cm). The CCTT‐2 administration involved connecting an alternating sequence of numbers (1–15) and colors (yellow/pink). We characterized the children's performance in the CCTT‐1 and CCTT‐2 using specific variables (see Table 2).

**TABLE 2:** Description of the CCTT‐1 and CCTT‐2 parameters

Parameters	Description	CCTT‐1	CCTT‐2
Completion test time (in seconds)	Total time to finish the test.	✓	✓
Difference Interference Index	Total completion time of the CCTT‐2—Total completion time of the CCTT‐1/Total completion time of the CCTT‐1	✓	✓
Number of errors	The number of times the respondent connects a circle in an improper numerical sequence	✓	✓
Number of near‐misses	The number of times that the respondent initiates a line towards an incorrect circle and they self‐correct	✓	✓
Number of prompts	The number of times that the tester points to the correct circle after the respondent delays the initiation of line drawing for more than 10 s	✓	✓
Number of color errors	The number of times the respondent incorrectly connects a circle with an improper color		✓

#### Verbal fluency

Fluency abilities were tested with the semantic and the phonemic fluency tests. In semantic fluency, participants were asked to verbally generate as many words from the semantic categories of animals, fruit and objects, allowing 1 min for each category. There were four dependent measures: the total number of correct words produced, as well as the number of correct words in each semantic category (animals, fruit, objects). The instructions that were provided to the children for the semantic fluency task were as follows: “When you hear the word” fruit, “what fruit comes to your mind? Tell me, in one minute, all the words that come to your mind.” Before the evaluation, the experimenter made sure that the child has understood the instructions by testing her/his performance on the category of professions, which was used as an example. If the child responded correctly to the example category, the experimenter praised the child and testing continued from there. Regarding the objects category, children's answers mainly consisted of clothing, musical instruments, toys, furniture, school supplies and transports.

In the phonemic fluency test, children were asked to verbally generate as many words as possible beginning with letters A /a/, S /s/ and X /ç/, allowing 1 min for each letter (Kosmidis et al., 2004). There were four dependent measures: the total number of correct words produced, as well as the number of correct words in each letter category (A /a/, S /s/, X /ç/). The phonemic verbal fluency task focused on the actual phoneme that each word started with and the instructions were as follows: “Please state, in one minute, all the words that come to your mind, that begin with the following phoneme.” Before the evaluation, the examiner made sure that the child has understood the instructions by testing her/his performance on the test phoneme, P /p/. If the child responded correctly to the test phoneme, the experimenter praised the child and testing continued from there. Children were also instructed that they could not use names of people, places, and forms of the same word (e.g., salata/salates [salad/salads]).

### Analysis plan

Because the predictors of the experiments' performance data were categorical and to cope with the unequal group sizes and sex ratios in each group of the study, the statistical analyses involved logit mixed effects regression models taking Group (TD, autism), sex (male, female), VIQ and PIQ as fixed effects, and including crossed random intercepts and slopes for participants (Baker, 2022; Jaeger, 2008). The models were performed separately for the CCTT‐1, CCTT‐2, the semantic and the phonemic fluency tests.

The dependent measures in the CCTT‐1 and CCTT‐2 were completion time, errors, near misses and prompts, while an additional variable for the CCTT‐2 was color errors. Analyses were also conducted on the CCTT difference interference index.

In the semantic fluency test, dependent measures were the total number of correct words produced, as well as the number of correct words in each semantic category (animals, fruit, objects). In the phonemic fluency test, dependent measures were the total number of correct words produced, as well as the number of correct words in each letter category (A /a/, S /s/, X /ç/). Besides mixed effects regression models, pairwise *t*‐tests for each group were conducted between categories separately for the semantic and the phonemic fluency test. Also, semantic and phonemic errors in the semantic and the phonemic fluency test, respectively, were measured and compared between groups using one‐way ANOVA analyses. Due to the fact that the children provided few responses in each verbal fluency task, no analyses were conducted on cluster sizes and switches (see the Appendix for the descriptive values of each group's clustering and switching data in the verbal fluency tests, Table A1).

Age, sex, VIQ and PIQ were included as predictors in all models. The models were fitted in R using the lmer function from the lme4 package (Bates & Maechler, 2009).

## RESULTS

Table 3 displays each experimental group's means and standard deviations for each task.

**TABLE 3:** Experimental groups' performance means (SDs) on the CCTT‐1, CCTT‐2, semantic fluency and phonemic fluency tests

Task	Experimental group	
	TD (*n* = 200)	Autism (*n* = 35)
CCTT‐1		
Completion time	54.6 (*17.8*)	56.80 (*25.9*)
Errors	0.16 (*0.4*)	0.27 (*0.7*)
Near misses	0.56 (*0.9*)	0.88 (*1.2*)
Prompts	0.18 (*0.5*)	0.33 (*1.06*)
CCTT‐2		
Completion time	92.36 (*26.7*)	114.60 (*43.6*)
Color errors	0.53 (*0.7*)	1.80 (*1.7*)
Number errors	0.17 (*0.4*)	0.47 (*0.7*)
Near misses	0.52 (*0.8*)	1.08 (*1.3*)
Prompts	0.43 (*0.6*)	0.66 (*1.1*)
Difference interference index (CCTT2 Time raw score‐CCTT1 Time raw score/CCTT1 Time raw score)	0.81 (*0.6*)	1.30 (*0.9*)
Semantic fluency		
Total	25.41 (*6.2*)	21.36 (*5.5*)
Animals	9.55 (*2.9*)	9.66 (*3.4*)
Fruit	7.21 (*1.9*)	6.80 (*3.0*)
Objects	8.73 (*3.2*)	4.88 (*2.3*)
Phonemic fluency		
Total	13.16 (*5.6*)	9.41 (*2.1*)
A /a/	4.86 (*2.2*)	3.52 (*0.9*)
S /s/	4.24 (*2.4*)	3.27 (*1.1*)
X /ç/	4.08 (*2.1*)	2.61 (*1.0*)

We first analyzed the effect of Group (TD, autism) on children's performances in the CCTT separately for the CCTT‐1 and CCTT‐2 component. In the CCTT‐1, there was a significant age effect in the category of near misses, which was due to the fact that the older children tended to initiate an incorrect circle and then self‐correct considerably fewer times than the younger children (see Table 4). For the CCTT‐2, the mixed effects model showed a significant main effect of Group on Completion time, which stemmed from the fact that the autistic children required more time to complete the CCTT‐2 task compared to the TD group. Furthermore, there was a significant Group effect on the color error measure, since the autistic children tended to incorrectly connect a circle with an improper color significantly more times than their TD peers (see Table 5). Finally, a significant difference between groups for the CCTT Interference Index was found, since the autistic children exhibited greater interference than the TD group in the CCTT‐2 (see Table 6). Finally, according to the mixed effects regression models, there were no significant effects for sex, VIQ, and PIQ.

**TABLE 4:** Summary of logit mixed effects model for the CCTT1: Completion time, errors, near misses and prompts

Predictors	Completion time				Errors				Near misses				Prompts			
	Coefficient	SE	*z*	*p* Value	Coefficient	SE	*z*	*p* Value	Coefficient	SE	*z*	*p* Value	Coefficient	SE	*z*	*p* Value
Intercept	149.55	44.08	3.39	0.660	0.77	1.08	0.71	0.915	8.07	2.26	3.57	0.096	2.22	1.49	1.49	0.506
Group	−5.57	44.25	−0.12	0.923	−1.13	1.09	−1.05	0.751	−3.74	2.28	−1.64	0.202	0.53	1.44	0.36	0.750
Age (in months)	−1.19	0.54	−2.18	0.694	−0.01	0.01	−0.52	0.918	−0.09	0.03	−3.35	0.047*	−0.03	0.02	−1.37	0.494
Sex	113.82	40.26	2.82	0.942	0.07	1.21	0.06	0.997	3.24	2.33	1.38	0.981	0.78	1.39	0.55	0.958
VIQ	−3.01	10.39	−2.91	0.772	−12.91	15.98	−0.80	0.420	−19.91	15.98	−1.24	0.214	1.58	20.63	0.08	0.939
PIQ	−11.17	21.60	−0.52	0.606	−0.50	0.51	−0.98	0.329	−0.67	0.72	−0.92	0.357	0.17	0.37	0.45	0.656

*Note*: SE, standard error; VIQ, verbal IQ; PIQ, performance IQ; Group levels: typically‐developing versus autism; Reference level for Group: autism.

* *p* < 0.05.

**TABLE 5:** Summary of logit mixed effects models for the CCTT2: Completion time, color errors, number errors, near misses and prompts

Predictors	Completion time				Color errors				Number errors				Near misses				Prompts			
	Coefficient	SE	*z*	*p* Value	Coefficient	SE	*z*	*p* Value	Coefficient	SE	*z*	*p* Value	Coefficient	SE	*z*	*p* Value	Coefficient	SE	*z*	*p* Value
Intercept	229.13	68.05	3.36	0.814	11.32	2.03	5.56	0.904	4.33	1.01	4.29	0.387	2.60	1.65	1.57	0.959	0.67	1.43	0.47	0.932
Group	−80.42	1.12	−71.37	0.007**	−79.78	0.66	−89.99	0.008*	−3.82	1.20	−3.17	0.395	1.85	1.71	1.07	0.922	−1.24	1.47	−0.84	0.700
Age in months	−1.62	0.83	−1.94	0.821	−0.13	0.02	−5.08	0.895	−0.05	0.01	−3.98	0.359	−0.02	0.02	−1.15	0.968	−0.01	0.02	−0.13	0.976
Sex	−52.30	68.04	−0.76	0.955	−0.65	2.19	−0.29	0.991	0.02	0.04	0.54	0.760	−0.03	0.02	−1.21	0.826	0.04	0.05	0.83	0.429
VIQ	−5.04	16.50	−0.31	0.760	17.90	17.95	0.997	0.320	11.37	25.39	0.44	0.655	8.78	19.67	0.45	0.656	11.48	20.73	0.55	0.580
PIQ	−2.0	40.18	−0.5	0.960	−0.16	0.72	−0.23	0.818	0.25	0.37	0.67	0.503	−2.33	1.52	−1.53	0.128	0.25	0.66	0.38	0.706

*Note*: SE, standard error; VIQ, verbal IQ; PIQ, performance IQ; Group levels: typically‐developing versus autism; Reference level for Group: autism.

* *p* < 0.05;

** *p* < 0.01.

**TABLE 6:** Summary of logit mixed effects models for the difference interference index in the CCTT task

Predictors	Completion time			
	Coefficient	SE	*z*	*p* Value
Intercept	1.05	0.06	16.61	<0.001***
Group	−0.25	0.07	−3.93	<0.001***
Age in months	0.02	0.03	0.45	0.653
Sex	0.03	0.07	0.44	0.706
VIQ	2.02	15.92	0.13	0.899
PIQ	0.06	0.95	0.07	0.948

*Note*: SE, standard error; VIQ, verbal IQ; PIQ, performance IQ; Group levels: typically developing versus autism; Reference level for Group: autism.

*** *p* < 0.001.

For the semantic fluency tests, the mixed effects model showed significant main effects of Group and age in the total number of items across the semantic subcategories. The autistic children tended to produce a lower amount of words compared to the TD group; also, the older children were found to produce more words than the younger ones. When split by semantic category, however, the analyses showed that the Group effect mainly stemmed from the Objects category (see Table 7). Within‐group paired t‐tests revealed that, for the TD group, the items generated in the fruit category were significantly fewer as compared to the animals, *t*(199) = 7.577, *p* < 0.001, and the objects, *t*(199) = 4.235, *p* < 0.001, while object words were significantly fewer than animal items, *t*(199) = 2.516, *p* = 0.013. For the autistic group, the items generated in the object category were significantly fewer as compared to the animals, *t*(35) = 8.353, *p* < 0.001, and the fruit, *t*(35) = 2.981, *p* = 0.005, while fruit words were significantly fewer than animal items, *t*(35) = 3.674, *p* = 0.001. Furthermore, the autistic children (mean semantic error rate: 4.7 [*1.5*]) tended to produce significantly more semantic errors in the semantic fluency test as compared to the TD group (mean semantic error rate: 1.6 [*1.0*]), *F*(235) = 198.005, *p* < 0.001.

**TABLE 7:** Summary of logit mixed effects models for semantic fluency: Total accuracy, and accuracy for the animals, fruit and object category

Predictors	Total accuracy				Animals				Fruit				Object			
	Coefficient	SE	*z*	*p* Value	Coefficient	SE	*z*	*p* Value	Coefficient	SE	*z*	*p* Value	Coefficient	SE	*z*	*p* Value
Intercept	23.38	0.57	40.39	<0.001	−8.47	7.13	−1.18	0.399	−3.52	5.49	−0.64	0.637	6.79	0.29	23.17	<0.001***
Group	2.02	0.57	3.50	<0.001***	−1.39	7.64	−0.18	0.910	2.07	5.59	0.37	0.800	1.93	0.32	1.31	0.046*
Age (in months)	0.49	0.14	3.47	0.003**	0.22	0.08	2.57	0.103	0.13	0.07	1.93	0.274	0.13	0.09	1.42	0.351
Sex	−1.58	15.07	−0.10	0.996	−9.08	7.36	−1.23	0.989	11.21	6.36	1.76	0.116	−1.18	7.46	−0.16	0.999
VIQ	5.33	5.12	1.04	0.301	2.00	4.96	0.40	0.688	1.00	6.28	0.16	0.874	3.10	5.25	0.59	0.556
PIQ	6.00	8.74	0.68	0.494	2.33	4.63	0.43	0.667	1.90	1.83	1.03	0.305	1.21	1.76	0.69	0.493

*Note*: SE, standard error; VIQ, verbal IQ; PIQ, performance IQ; Group levels: typically‐developing versus autism; Reference level for Group: autism.

* *p* < 0.05;

** *p* < 0.01;

*** *p* < 0.001.

In the phonemic fluency test, the autistic group was found to produce overall fewer words compared to the TD group, however, when split by letter, the Group effect was found to be due to the groups' performances in the X /ç/ letter. Also, there was a significant age effect for the X /ç/ letter category, since the younger children tended to produce considerably fewer words beginning with X /ç/ than the older children (see Table 8). Within‐group paired t‐tests revealed that, for the TD group, the items generated in the X /ç/ letter category were marginally significantly fewer as compared to the S /s/ words, *t*(199) = 1.966, *p* = 0.053, and significantly fewer the A /a/ items, *t*(199) = 3.618, *p* < 0.001, while A /a/ words were significantly more than S /s/ words, *t*(199) = 2.698, *p* = 0.008. For the autistic group, the items generated in the X /ç/ letter category were significantly fewer than both the S /s/, *t*(35) = 3.095, *p* = 0.004, and the A /a/ items, *t*(35) = 3.416, *p* = 0.002. The difference between the S /s/ and the A /a/ items was not statistically significant, *t*(35) = 0.475, *p* = 0.638. The two groups did not differ in their phonemic error rates (mean phonemic error rate for the autistic children: 0.5 (*0.7*), mean phonemic error rate for the TD children: 0.4 (*0.6*)), *F*(235) = 0.927, *p* = 0.337. Finally, according to the mixed effects regression models, neither sex nor VIQ‐PIQ reached significance in either the semantic or the phonemic fluency test.

**TABLE 8:** Summary of logit mixed effects models for phonemic fluency: Total accuracy, and accuracy for the letters a /a/, S /s/, X /ç/

Predictors	Total accuracy				Letter A /a/				Letter S /s/				Letter X /ç/			
	Coefficient	SE	*z*	*p* Value	Coefficient	SE	*z*	*p* Value	Coefficient	SE	*z*	*p* Value	Coefficient	SE	*z*	*p* Value
Intercept	11.29	0.48	23.58	<0.001	−1.04	3.71	−0.27	0.782	2.63	4.03	0.65	0.515	3.34	0.18	18.59	<0.001***
Group	1.87	0.47	3.91	<0.001***	−3.36	3.84	−0.87	0.435	−6.11	4.07	−1.50	0.145	−7.64	3.99	−1.91	<0.001***
Age (in months)	0.15	0.12	1.30	0.198	0.07	0.05	1.42	0.165	0.02	0.05	0.29	0.769	0.13	0.05	2.31	0.048*
Sex	−7.65	13.73	−0.55	0.998	−1.60	4.61	−0.34	0.999	−4.28	5.30	−0.81	0.999	0.59	4.38	0.13	0.998
VIQ	6.33	3.99	1.58	0.116	4.87	3.87	1.26	0.210	2.00	4.89	0.41	0.684	2.64	3.70	0.71	0.477
PIQ	4.83	8.79	0.55	0.584	−1.16	1.92	−0.61	0.547	1.33	1.12	1.18	0.241	1.75	0.94	1.84	0.108

*Note*: SE, standard error; VIQ, verbal IQ; PIQ, performance IQ; Group levels: typically developing versus autism; Reference level for Group: autism.

* *p* < 0.05;

*** *p* < 0.001.

## DISCUSSION

The current study aimed to investigate the cognitive flexibility skills of autistic children and age‐, VIQ‐, PIQ‐, and SES‐matched TD children by administering the CCTT and verbal fluency tests, both purported to measure cognitive flexibility. Autistic individuals have been shown to encounter difficulties in cognitive flexibility, such as difficulty switching from one task or perspective to another in both verbal and non‐verbal experimental paradigms (Eigsti et al., 2008; Mostert‐Kerckhoffs et al., 2015; Ozonoff et al., 2004; Peristeri et al., 2021; Remington & Fairnie, 2017). However, little work has explored cognitive flexibility as an appropriate context to characterize the cognitive profile of younger autistic children. It is thus unclear whether young autistic children exhibit difficulties in cognitive flexibility relative to their neurotypical peers, or group differences rather stem from task demands and variability. According to the results of the current study, the autistic children were found to perform poorer compared to their TD peers only on the CCTT‐2 component that tapped into set‐shifting, whereas on the CCTT‐1 component, that required no attention switching, both groups achieved a similar performance. In the fluency tasks, the autistic children performed worse than neurotypical children in semantic and phonemic fluency, yet, such difference stemmed from the autistic group's drop of performance in specific categories of the tasks, namely, in the object and the X /ç/ letter category of the semantic and the phonemic fluency task, respectively. The overall evidence shows a robust effect of autism on the cognitive flexibility skills of young autistic children as manifested in the nonverbal CCTT‐2 task. However, the children's fluency performance seemed to be driven selectively by language‐specific properties of the semantic and phonemic tasks, which further suggests that the young autistic children's fluency performance did not tap into their cognitive flexibility skills only, but rather relied on a complex balance of cognitive flexibility and features of the linguistic stimuli.

Specifically, in the CCTT task, autistic children were significantly slower on the CCTT‐2 than typically‐developing children, pointing to reduced cognitive flexibility skills for the autistic group. For the CCTT‐1, there was no difference between groups, which is in line with previous studies demonstrating that children with and without autism have similar cognitive efficiency profiles as measured by processing speed. Specifically, autistic individuals have been found to show decreased sensitivity to the perception of complex information, whereas their integration of simple information is either intact (Bertone et al., 2003) or even superior (Bertone et al., 2005). On the other hand, compared to the neurotypical group, the young autistic children exhibited slower performance in the color error measure of the CCTT‐2, which suggests reduced cognitive flexibility skills. For the CCTT Interference Index, the difference between the two groups was also significant, and strengthens the evidence that the autistic children had difficulties in cognitive flexibility. Age was found to influence both autistic and neurotypical groups' near‐misses, which were associated with the children's error monitoring and self‐correction abilities. It seems that younger children across both groups had difficulty with detecting mismatching links between circles and colors in the CCTT‐2 as compared to the older children, which may in part be attributed to age‐related changes in processing speed or/and developmental changes in the children's executive functioning control skills.

Moving on to the performance in the fluency tasks, group differences seemed to be dependent on specific functions of the linguistic stimuli rather than to domain‐general difficulties in cognitive flexibility. Specifically, the autistic children appeared to have generated overall fewer word items than their neurotypical peers in the semantic fluency task, yet, this group difference seemed to stem from the autistic children's selective drop of performance in the object category only. Qualitatively, the pattern for object word generation in the autistic children was different from TD controls, since objects for the autistic group was the category with the fewest items generated in the semantic fluency test, while object words for the TD group were significantly more than fruit words.

The different pattern of results observed for the objects versus animals & fruit semantic categories suggests that the group effect was driven by the lexical knowledge pertaining to the semantic category of objects and the efficiency with which the autistic children updated their mental lexicon in their active search for words denoting objects. There is literature showing that young autistic children show reduced sensitivity to different semantic‐conceptual properties of objects, such as shapes and functions, that provide the basis for word learning in TD children. For instance, Field et al. (2016) found that 9‐year‐old autistic children found function bias considerably less informative for lexical extension of new words denoting objects as compared to a group of TD age‐matched children. Similarly, shape biases in object word learning were not found to contribute to autistic children's ability to update their predictions regarding the semantic classification of novel words referring to objects (Potrzeba et al., 2015). If autistic children use different mechanisms for capturing form/function and meaning correspondences that shape the lexicon to converge on discrete semantic categories, then they may be less likely to constrain lexical choices to enable the production of semantically related words. Especially the category of objects constitutes a rather broad semantic class that encompasses a large variety of possible lexical entries whose attributes tend to be conceptually arbitrary in children's non‐linguistic representations of objects (Bion et al., 2013; Hollich et al., 2007). Given the difficulties in executive functioning, rigidity and perseverative thought and behavior observed in autistic individuals (Hill, 2004), difficulties in recognizing the boundaries of the category of objects may be expected. In addition, autism is also associated with high rates of anxiety, which is often characterized by hypervigilance to less familiar stimuli (Peled‐Avron & Shamay‐Tsoory, 2017); autistic children in the current study might have been reluctant to offer answers in a category that would cost their overall accuracy. The results suggest that conceptual properties specific to the words denoting objects, rather than difficulties in cognitive flexibility, may have driven autistic children's performance drop for the specific category in the semantic fluency task.

Word category‐specific performance drops were also observed in the phonemic fluency task. The young autistic children have generated significantly fewer word items in the category of the X /ç/ letter relative to the A /a/ and S /s/ letter categories. Similarly, neurotypical controls also produced fewer lexical items in the X /ç/ letter category relative to the S /s/ and the A /a/ item category. We should note that velar fricatives such as X /ç/ in Greek have the lowest spectral mean in terms of the duration and the intensity of frication (Nirgianaki, 2014), and autistic individuals hear with poor spectral resolution (Boets et al., 2015; Oram Cardy et al., 2005). Relevant research has shown that autistic children exhibit severe auditory perceptual difficulties (Dunlop et al., 2016; Erviti et al., 2015) that may cause speech discrimination difficulties in particular sound contexts. However, an alternative explanation is that the low number of word items beginning with X /ç/ in the autistic group was simply caused by the particular phoneme's very low frequency in oral speech in Greek, as verified in a study of early word productions of young TD children (Nicolaidis et al., 2003) and even in a corpus study with Greek adults (Hatzigeorgiu et al., 2000). If autistic children's difficulty with retrieving words beginning with X /ç/ reflects its frequency of occurrence in oral input, then we could argue that the drop in performance in the specific phoneme category was frequency‐dependent.

The overall results of the current study show that the autistic children had cognitive flexibility difficulties in the nonverbal CCTT measure, but they exhibited selective difficulties in generating words in specific categories, more specifically, in the object and the letter X /ç/ subcategories of the semantic and the phonemic fluency task, respectively. Interestingly, the autistic children made considerably more semantic errors than their TD peers in the semantic fluency test, while both groups' alphabetic error rates in the phonemic fluency test were very similar. These results hint at autistic children's difficulty with distinguishing between exemplars that belong to particular semantic categories which may stem from difficulties allocating attentional processes towards attributes of interest. In addition, the findings suggest that cognitive flexibility performance in autism may not only be related to domain‐general cognitive skills, but also to the children's language skills, once the cognitive flexibility measure is verbal, and to the features of the linguistic stimuli. The autistic group seemed to be able to cope with the fluency tasks and scored lower than their neurotypical peers only in specific subcategories possibly due to functional and frequency characteristics of the linguistic stimuli (i.e. object words and lexical items beginning with the letter X /ç/). This suggests that language in the fluency tasks may have affected the result pattern of the autistic children who presumably used different mechanisms than their neurotypical peers in coping with the verbal fluency tasks. Current research (Haebig et al., 2015; Weismer et al., 2018; see Friedman & Sterling, 2019 for a review) on cognitive correlates of language difficulties in autistic children shows that executive functions are positively correlated with language skills in the areas of lexical processing, structural and pragmatic language, though the direction of influence between the two domains remains unknown. Although more research is needed to shed light on the mechanisms behind the relationship between cognitive flexibility and language in autism, this study underlines the promising role of verbal cognitive flexibility paradigms in the study of the cognitive profile of young autistic children. Finally, the finding that neither VIQ nor PIQ significantly predicted autistic children's variance of performance in either the CCTT or the verbal fluency tests suggests that general intelligence quotient scores based on the WPPSI‐III are not associated with cognitive flexibility skills in young autistic children. Perhaps single index scores drawn from WPPSI's subtests might have shown more sensitivity to the children's performance in the cognitive flexibility tests as compared to overall intelligence scores.

Turning to the implications of this research, these can be considered at two levels. From a theoretical perspective, the findings may inform hypotheses about difficulties in cognitive flexibility in young autistic children and the way it can be affected by language. It seems that the autistic children's ability to shift and maintain new responses based on changing instructions was more preserved in the fluency tasks as compared to the nonverbal CCTT paradigm, attesting to the important role of language in experimental paradigms that aim at assessing cognitive skills in autism. A number of studies on the cognitive profiles of autistic children have pointed out the potential effects of language on the participants' performances, and the possibility that these tests measure the children's verbal abilities rather than their cognitive skills per se (Astington et al., 2002; Grueneisen et al., 2015; Peristeri et al., 2021). It would thus be helpful to have nonverbal tests of autistic children's cognitive skills, including cognitive flexibility. Of course, further research is needed to investigate individual variability and the interaction between the autistic children's linguistic skills and their cognitive flexibility performance in the current study. Importantly, autism effects on the cognitive flexibility skills of young children are also relevant for interventions in clinical settings at a practical level. The findings indicate that the CCTT is sensitive to young autistic children's difficulties in cognitive flexibility and could be used to provide a better understanding of cognitive difficulties in autism. Especially, considering the importance of early intervention, it is of great importance to find quick and easy ways to investigate the cognitive profiles of young autistic children. Tests such as the CCTT may allow clinicians to screen autistic children for difficulties in cognitive flexibility in a quick, engaging and efficacious manner. In addition, the results of the current study suggest that the autistic children's spoken language ability may be related to their performance in the verbal fluency tests; spoken language ability may, thus, have the potential to contribute to autistic children's improvement in cognitive flexibility. The finding that autistic children fell behind their TD peers on specific word categories of the fluency tests provides a hint at one of the possible ways to attenuate difficulties in cognitive flexibility in autism through enhancing the children's spoken language, such as vocabulary. More research should be conducted to explore the possible cognitive flexibility gains following spoken language ability intervention in autism.

The current study has the following limitations. First, we have not used any background language ability measures, such as vocabulary, that could have helped to elucidate more the results in the verbal fluency tests. Second, the small sample size of the autistic children that have participated in the current study may have limited the statistical power of the results, and, third, more measures which include a switching component could have shed more light on the evaluation of the autistic children's cognitive flexibility skills. Further studies are warranted to investigate cognitive flexibility competence in young autistic children.

## CONFLICT OF INTEREST

The authors declare no conflict of interest.

## ETHICS STATEMENT

All authors certify that they have no affiliations with or involvement in any organization or entity with any financial interest or non‐financial interest in the subject matter or materials discussed in this manuscript. The study was approved by the Research Ethics Committee of the Greek Ministry of Education (Ref. No.: HAA37HAA87). Written informed consent was obtained from the children's parents. The study was performed in accordance with the ethical standards as laid down in the 1964 Declaration of Helsinki.

## Data Availability

The data that support the findings of this study are available from the corresponding author upon reasonable request.

## Appendix

**TABLE A1:** Means (and standard deviations) of cluster sizes and switches by group, fluency test, and category, and total cluster sizes and switches by group and fluency test

Tasks	Measures	Experimental group	
		TD (*n* = 200)	Autism (*n* = 35)
Semantic fluency	Cluster size—Animals	1.28 (*0.5*)	1.27 (*0.4*)
	Cluster size—Fruit	1.26 (*0.4*)	1.28 (*0.4*)
	Cluster size—Objects	1.68 (*0.7*)	1.38 (0.6)
	Total cluster size	4.24 (*1.1*)	3.94 (*0.9*)
	Switches—Animals	0.29 (*0.5*)	0.27 (*0.4*)
	Switches—Fruit	0.26 (*0.4*)	0.29 (*0.5*)
	Switches—Objects	0.93 (*0.8*)	0.38 (*0.6*)
	Total switches	1.53 (*1.7*)	0.94 (*1.0*)
Phonemic fluency	Cluster size—Letter A /a/	1.10 (*0.3*)	1.22 (*0.4*)
	Cluster size—S /s/	1.13 (*0.4*)	1.03 (*0.2*)
	Cluster size—Letter X /ç/	1.26 (*0.5*)	1.14 (*0.4*)
	Total cluster size	3.50 (*0.9*)	3.38 (*0.5*)
	Switches—Letter A /a/	0.11 (*0.3*)	0.22 (*0.4*)
	Switches—Letter S /s/	0.14 (*0.4*)	0.03 (*0.2*)
	Switches—Letter X /ç/	0.26 (*0.5*)	0.13 (*0.3*)
	Total switches	0.51 (*0.8*)	0.38 (*0.5*)

*Note*: Cluster size in semantic fluency = words that share the same semantic subcategory (e.g., jata [cat], skilos [dog] in animals). Cluster size in phonemic fluency = words that begin with same first two letters (alepu [fox], alojo [horse]), words that differ only by a sound (çina [goose], çira [widow]), rhyming words (soma [body], stroma [mattress]). Switches = number of transitions between clusters.
